# Editorial Correspondence

**Published:** 1879-11

**Authors:** John Westcott

**Affiliations:** Tocoi, Florida


### EDITORIAL CORRESPONDENCE.

TOCOI, FLORIDA, August 10th, 1879.

*J. G. Westmoreland, M.D.:*

*Dear Sir:* Your pamphlet on yellow fever from "Transactions Medical Association of Georgia," is received. I have read it with much pleasure. I am always pleased with minds that dare assault inconsistency, and dare go out of "old ruts" when they discover better. There is nothing too old to be disputed. The whole truth should always be told. There is as much in *being able* to discover the sources of error, as in discovering truth. I like the spirit of your pamphlet very much; with such minds co-operating, the infernalism of quarantine will fall.

I hope you will study my theory and criticise where it appears lame. Understanding, as you do, electricity, meterology, magnetism, physiology, psycology, and the atomic theory, you can get proper *facts.* How does the *lightning* of the clouds get up above the dew point? You will find the electrical condition of the atmosphere is *periodic*, rising from sunrise till about 3 P.M., and then rising slightly for a short time, until after sunset, and then falling until day light, and so on regularly, but controlled by circumstances. A sensitive instrument will show you the intensity. The amount of evaporation of water, in polarized molecules, is easily discoverable. By systematized study all these are discernable.

Drainage, thorough drainage, to prevent excessive evaporation will prevent the higher grades of fever, and fevers should be graded, corresponding to the intensity of the surrounding electrical atmosphere. This accounts for the cold, hot, sweaty or relief stage, and every other phenomena accompany every grade, even to black vomit and the yellow appearance or spots.

I am not engaged in the practice of medicine. A short time after the Seminole war, in which I served as regimental and brigade surgeon, I entered into other business, but have ever been a student of the natural sciences, and have kept myself up in medicine and ever saw the deficiency in medicine in not knowing the proximate and primary causes of fevers, which would account for all the phenomena presented.

The remedies are to equalize the nervous influence and destroy the disturbing cause. Make the molecules and nervous organization as strong or intense as the outside influences. Stimulants, if given properly, will do it. They may be given by an intelligent hand. The most successful practice, has been stimulants, *without knowing why*—experience taught it.

I have no faith that dry or moist "filth," as it is popularly understood, has anything to do with fevers. "Bad smells," dirty and unsightly yards, seem uncomfortable, and to well raised people are both disagreeable and uncomfortable—*and that is all.* Old cabbage leaves, water mellon shells, chicken feathers and bones, fish scales, etc., old bones, potato peelings, peach pits, apple cores, old shoes, scraps of paper mixed with weeds, etc., etc., lying about the yard loose, do not look very well, tidy or cleanly, but there is no *cause* of fever there. Smell don't amount to much. Disagreeable and pleasurable smells are educational and conventional. This may strike you with horror, but think of it philosophically : sour-krout and limburger cheese and chitlens for one; otto of roses and night-blooming cereus for another. The smell of musk-rat and beaver and ammonia in cut glass phials are tolerated in fashionable parlors—but not the polecat or the *ammonia* from the stable. So then when not in such quantities as to disturb the breathing arrangements, and supply of vital air, they will not produce mal-adjustment. But as there is

*no objection*, and it seems *innocently* popular, it does no harm *at present* to retain the idea that, with thorough drainage, as a *preventive*, fever may be controlled. Retain it, because we are taught that "cleanliness is next to godliness," and all know that it prevents parasites, not only on the head, but the body; and it is always well to get rid, and keep rid, of these CONTAGIOUS PESTS, that seem to originate in dirt and filthy habitations; and in armies there is an hypothesis that they may "grow" from the *silaccous dust*, vitiated by the magnetic power of the nerves of the organization.

I have but one axe to grind in this whole matter; and it is fair and legitimate: that is, to get credit for helping medicine to a "CERTAIN SCIENCE" in fevers, *if nothing more.* If I can do it, I am as much entitled to this credit as was Franklin, Faraday, Davy and Priesley—in fact all scientists who have discovered the application of a principle; and nothing would please me better than to have scientists give my theory a fair and just criticism, and upon the rule *that modern science recognizes no such thing as an exception to any natural law.*

Knowledge, of course, affects and excites our admiration, calms our fears, and destroys the theological scholastic *superstitions* and teachings of former years. Science does not acknowledge, under this rule, and cannot, the theological and scholastic definition of *miracle*—that "a miracle is a suspension of a law of nature." Every operation that has or will transpire has and will be governed by natural laws.

The scholastic philosophers, when they could not understand anything, referred it to moral causes. There is now as much learning, out of the priesthood, as there is or ever was in it—and men that understand theology and the natural sciences equally well.

It has been different. St. Augustine and Cardinal Bonafice would not admit the world to be round, because they could find no *provision in the Scripture* for the salvation of the inhabitants, if there was any, on the opposite side of the sphere, and prohibited its teaching. So it has been with the world; and so it has been with diseases. *Superstition* has been encouraged, and is now.

This letter was not prepared for publication; otherwise disseminate the theory as much as you please. We are strangers, but I hope you will not "cut" me on account of my "little" doctrine until you examine it and give me a chance to redeem myself for any faults that may be demonstrated. Respectfully yours,

JOHN WESTCOTT.

